# Clinically inactive disease in juvenile dermatomyositis – a proposed revision to the printo criteria

**DOI:** 10.1186/1546-0096-12-S1-P88

**Published:** 2014-09-17

**Authors:** Beverley Almeida, Katie Arnold, Raquel Campanilho-Marques, Lucy Wedderburn, Clarissa Pilkington, Kiran Nistala

**Affiliations:** 1 Department of Rheumatology, Great Ormond Street Hospital for Children NHS Trust, UK; 2 Infection, Inflammation and Rheumatology Section, University College London Institute of Child Health, UK; 3 Arthritis Research UK Centre for Adolescent Rheumatology, UCL, UCLH, GOSH NHS Trust, UK; 4 Centre for Rheumatology, University College London, London, UK

## Introduction

Juvenile dermatomyositis (JDM) affects 3 children/million/year with myositis and skin disease being the typical features. PRINTO have recently established criteria to classify JDM patients who are clinically inactive by meeting at least 3 out of the following 4 conditions – CK ≤150, CMAS ≥48, MMT8 ≥78 and physician global VAS (PGA) ≤0.2. CK, CMAS and MMT8 all measure muscle involvement, only PGA includes skin or other organ involvement. The hypothesis that these criteria may fail to detect patients who have active skin disease but normal muscle parameters was tested.

## Objectives

To demonstrate the prevalence of clinically inactive disease in the UK JDM Cohort and Biomarker Study and to identify whether skin disease is still present in these patients on the basis of the PRINTO criteria.

## Methods

Data were analysed from children who were recruited and met Bohan-Peter criteria. Data from patient episodes (either a clinic visit or hospital admission) were assessed using the PRINTO criteria. Using the PRINTO rules stipulating 3 of 4 criteria are required, all data entries were divided into 2 groups based on the criterion that was omitted. Each case was analysed to determine whether skin disease was present or absent.

## Results

682 data entries (DE) from 321 patients were identified as clinically inactive. 255 (37.4%) of these DE (119 patients) met all 4 criteria. 21.2% of DE had skin rash and 10.5% had nailfold changes (Table 1) at the time of assessment. 427 of the total DE (202 patients) met 3 of the 4 criteria. Of these, 320 (74.9%) had clinically inactive based on the 3 muscle criteria (PGA was not met). 61.6% of this group had ongoing skin rash present. Among the 107 remaining DE, which were clinically inactive by 3 criteria of which one was PGA, the frequency of skin changes was lower. The differences between the 3 groups were statistically significant in terms of rash (χ_2_ 111.5, p<0.0001), nailfold changes (χ_2_ 65.5, p<0.0001) and calcinosis (χ_2_ 22.07, p<0.0001).

**Table 1:** Frequency of skin changes in JDM patients meeting PRINTO criteria (number of episodes, % in brackets)

Criteria met (DE=data entries, n=number of patients)	Rash	Nailfold changes	Calcinosis
All 4 criteria met (255 DE, n 119)	54 (21.2)	27 (10.5)	19 (7.5)

3 criteria met, but PGA not met (320 DE, n 131)	197 (61.6)	114 (35.6)	60 (18.8)

3 criteria met of which one was PGA (107 DE, n 71)	25 (23.3)	9 (8.4)	6 (5.6)

## Conclusion

This study is one of the first to test the PRINTO criteria in a large independent cohort of JDM patients. When clinically inactive disease is defined by “muscle-based” criteria, without PGA, there is a greater frequency of skin disease. As a revision, we propose that PGA should be included as an essential criterion together with 2 of the 3 muscle criteria. This would prevent skin disease being overlooked in the clinical assessment which is important since it is often recalcitrant to treatment and may be associated with poor long-term disease outcomes. A revision of the criteria would need testing in independent cohorts.

## Disclosure of interest

None declared.

